# Optic disc edema during strict 6° head-down tilt bed rest is related to one-carbon metabolism pathway genetics and optic cup volume

**DOI:** 10.3389/fopht.2023.1279831

**Published:** 2023-10-27

**Authors:** Sara R. Zwart, Brandon R. Macias, Steven S. Laurie, Connor Ferguson, Claudia Stern, Alex Suh, M. Mark Melin, Millennia Young, Eric Bershad, Scott M. Smith

**Affiliations:** ^1^ Human Health and Performance Directorate, University of Texas Medical Branch (UTMB), Galveston, TX, United States; ^2^ National Aeronautics and Space Administration (NASA), Johnson Space Center, Human Health and Performance Directorate, Houston, TX, United States; ^3^ Human Health and Performance Directorate, KBR, Houston, TX, United States; ^4^ Human Health and Performance Directorate, Aegis Corp, Houston, TX, United States; ^5^ Department of Clinical Aerospace Medicine, German Aerospace Center (DLR), Cologne, Germany; ^6^ Tulane University School of Medicine, New Orleans, LA, United States; ^7^ Gonda Vascular Center, Mayo Clinic, Rochester, MN, United States; ^8^ Neurology Department, Baylor College of Medicine, Houston, TX, United States

**Keywords:** optic disc edema, ocular, spaceflight, one-carbon metabolism, optic cup volume

## Abstract

Some astronauts on International Space Station missions experience neuroophthalmological pathologies as part of spaceflight associated neuro-ocular syndrome (SANS). Strict head-down tilt bed rest (HDTBR) is a spaceflight analog that replicates SANS findings and those who had 3–4 risk alleles (G and C alleles from the methionine synthase reductase [MTRR] A66G and serine hydroxymethyltransferase [SHMT1] C1420T, respectively) as compared to 1-2 risk alleles, had a greater increase in total retinal thickness (TRT). The objective of this study was to identify factors that contribute to the individual variability of the development of SANS in a 60 d HDTBR at the German Aerospace Center’s:envihab facility, Cologne Germany. 22 of 24 subjects who participated in the HDTBR study provided blood samples for genetic analysis. Total retinal thickness and optic cup volume were measured before and after bed rest. Subjects with 3–4 versus 0-2 risk alleles had greater ΔTRT during and after bed rest, and the model improved with the addition of baseline optic cup volume. This bed rest study confirms that variants of MTRR and SHMT1 are associated with ocular pathologies. Subjects with more risk alleles had the greatest HDTBR-induced ΔTRT, reaffirming that genetics predispose some individuals to developing SANS. Preflight optic cup volume and genetics better predict ΔTRT than either one alone. Whether nutritional supplements can override the genetic influences on biochemistry, physiology, and pathophysiology remains to be tested. These findings have significant implications for both aerospace and terrestrial medicine.

## 1 Introduction

Some astronauts on International Space Station missions develop ophthalmic pathologies characterized as part of spaceflight associated neuro-ocular syndrome (SANS). The cause of SANS remains unknown, but environmental, dietary, anatomical, and genetics may be contributing factors (1–3).

Circulating folate and vitamin B_12_-dependent one-carbon metabolic pathway intermediates were higher in SANS-affected astronauts, even *before* flight. While these intermediates were higher, serum folate was lower during flight in these astronauts (4), suggesting the cause of SANS could be a functional B vitamin insufficiency.

Astronauts with specific single nucleotide polymorphisms (SNPs) in one-carbon pathway genes, namely, the G allele for methionine synthase reductase (MTRR) A66G and the C allele for serine hydroxymethyltransferase (SHMT1) C1420T had a higher incidence of SANS pathologies (e.g., optic disc edema, globe flattening, choroidal folds) (5, 6). The degree of optic disc edema in subjects exposed to head-down tilt bed rest (HDTBR) in a 0.5% CO_2_ environment was higher in subjects with more G and C alleles (i.e., risk alleles) for MTRR 66 and SHMT1 1420, suggesting an association (7).

In astronauts, anatomical differences including smaller preflight optic cup volume have also been associated with greater inflight increases in peripapillary total retinal thickness (TRT) (3). Here we report results from a 60-d HDTBR investigation that explored the role of one-carbon pathway genes and baseline optic cup volume on the development of optic disc edema.

## 2 Methods

“Artificial Gravity Bed Rest with European Space Agency” (AGBRESA), a 60-d, 6° strict HDTBR study was conducted at the German Aerospace Center (DLR): envihab facility. 24 subjects (16 men, 8 women) participated. Study design and TRT findings have been published (8–18). None of the data reported here, i.e., the relationship of optic disc edema to one-carbon pathway genetics or optic cup volumes, have been previously published.

The ΔTRT was previously reported in these subjects (18). Because there was no difference between groups exposed to either continuous (30 min) or intermittent (6 x 5 min with a rest of 5 min in between) artificial gravity daily or controls, all subjects were pooled in the present analyses. The studies were approved by the NASA Institutional Review Board and the Medical Association of the North Rhine (Aerztekammer Nordrhein). Written informed consent was obtained from all subjects.

### 2.1 Genetic analyses

Blood samples were collected from 22 subjects at the 1-yr follow up session. Blood samples were frozen at -80°C until analyses. DNA extraction and SNP analyses for variants MTRR A66G (rs1801394) and SHMT1 C1420T (rs1979277) were performed as previously described (7).

### 2.2 TRT, RNFL, and chorioretinal folds

Optical coherence tomography (OCT, Spectralis Flex Module OCT2, Heidelberg, Germany) images were obtained from subjects in the supine position 6 d before, during, and up to 12 d after HDTBR. OCT images and TRT, retinal nerve fiber layer (RNFL) thickness, and folds quantification have been reported (18).

### 2.3 Blood biochemistry

Blood samples for biochemistry were collected and analyzed as previously described (8).

### 2.4 Statistical approach

As done previously (7), we categorized high and low risk based on the number of risk alleles in 2 SNPs of interest within the MTRR 66 and SHMT1 1420 genes. ΔTRT was compared between genetics groupings. Briefly, ΔTRT was analyzed through a mixed effects model defined in terms of the interaction of genetic grouping and time as categorical fixed effects. Nested subject and eye random effects addressed the repeated measures within individual eyes clustered within subjects. Robust standard errors addressed non-homogenous variance between genetic groupings. Differences were estimated and tested through expected marginal means. Further, ΔTRT was compared with the previous bedrest study by adding Study (AGBRESA or VaPER) to the interaction term of the model.

Optic cup volume was similarly modeled but the Gamma distribution was used to model the response since cup volume is positively bound.

Given the limited sample size, and need to quantify and interpret any effects, a Bayesian mixed model approach was also implemented. Gamma and exponential distributions were considered but resulting diagnostics were unacceptable. Therefore, we used a normal distribution for the cup volume variable within the Bayesian analysis. Diffuse inverse gamma priors were used for the nested subject and eye random effects and scale parameters, and a standard normal distribution for the fixed effects coefficients. The primary inference of interest for this approach was quantifying the posterior probability that the higher risk allele grouping had smaller mean cup volumes. We also analyzed the occurrence of folds through Bayesian logistic regression to quantify a similar probability, the posterior probability that the more risk allele group is associated with higher odds of developing folds. Bayesian and Frequentist mixed models were fit in SAS v9.4 with the GLIMMIX and BGLIMM procedures.

To determine whether genetic grouping modified the relationship between smaller pre-HDT cup volume and HDT-related ΔTRT, we conducted a likelihood ratio test between two models fit using maximum likelihood within R. The baseline model considered pre-HDT cup volume as the covariate, and ΔTRT as the dependent variable. The comparative model added genetic grouping as both an intercept, as well as slope (interaction with pre-HDT cup volume) modifier. Models were fit using maximum likelihood using the lme function within the lmer package. The anova function compared the two models to test for model improvements with inclusion of genetic grouping.

## 3 Results

**Figure 1:**
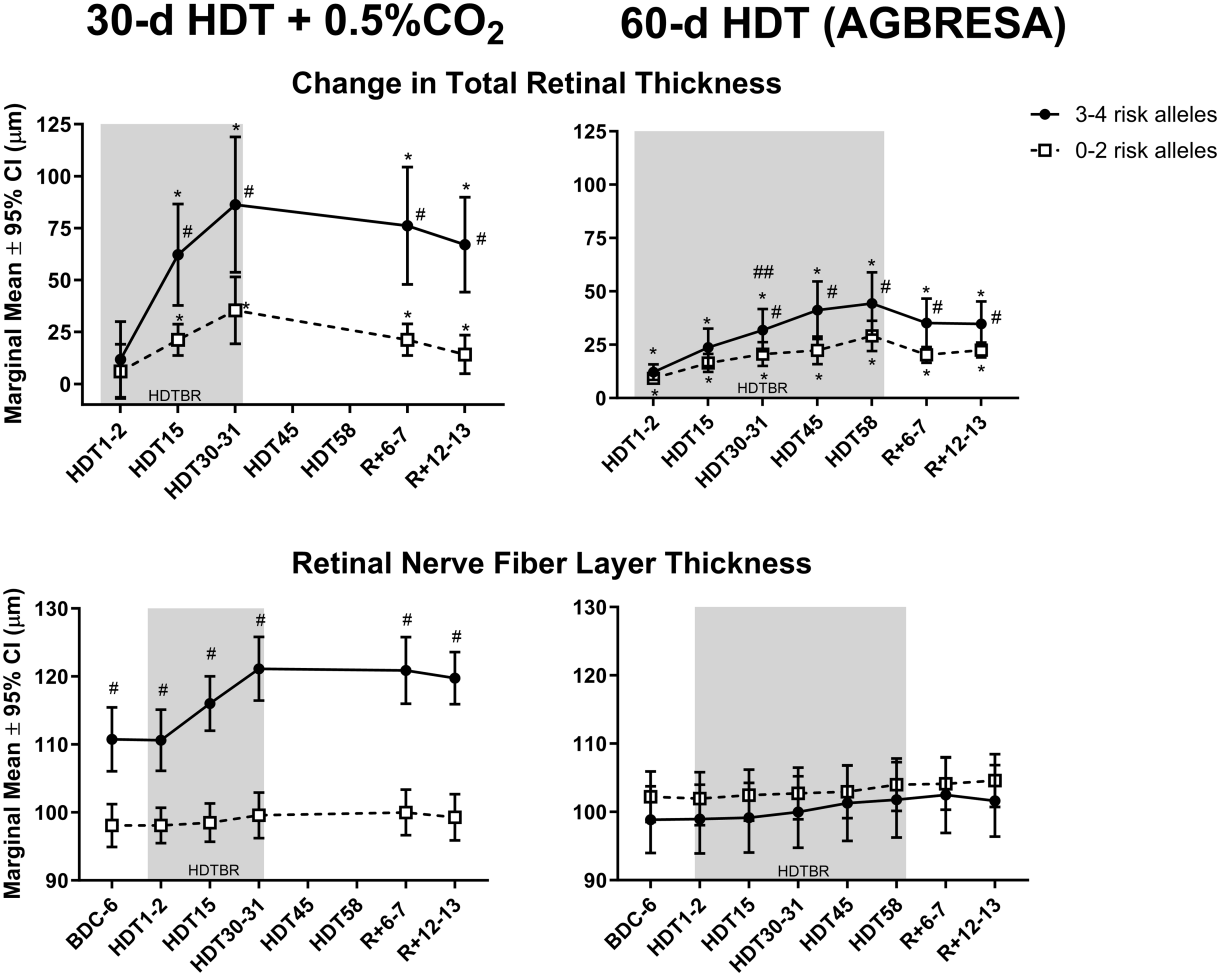
The top graphs depict mean (± 95% CI) change in peripapillary total retinal thickness in Artificial Gravity Bed Rest with European Space Agency (AGBRESA) subjects (right panel) with 3–4 (n=9) or 0–2 (n=13) risk alleles, after 2, 15, 31, 45, and 58 days of head-down tilt bed rest (HDTBR) and 6 and 13 days of recovery (R+6–7). Previously published 30-d HDTBR study data where subjects were exposed to 0.5% CO_2_ are included for comparison (7) (left panels). The mean thicknesses of the peripapillary retinal nerve fiber layer are presented in the bottom panels. Shading indicates the HDTBR phase of the study. ^#^Significantly different from subjects with 0–2 risk alleles (*P* <.05); *Significantly different from baseline total retinal thickness (*P* <.001); ^##^3–4 risk allele group significantly different from 3–4 risk allele group in the 30-d HDTBR + 0.5% CO_2_ bed rest study (p = 0.01).

The magnitude of ΔTRT in the region 250 µm from Bruch’s membrane opening was significantly greater in the group with 3-4 risk alleles (**Figure 1**, upper panels). That is, ΔTRT grouped by genetic category (0–2 vs 3–4 risk alleles) was significantly different after 31 d of HDTBR and persisted 12 d after HDTBR. RNFL did not increase during HDTBR in either genetic category.

**Figure 2:**
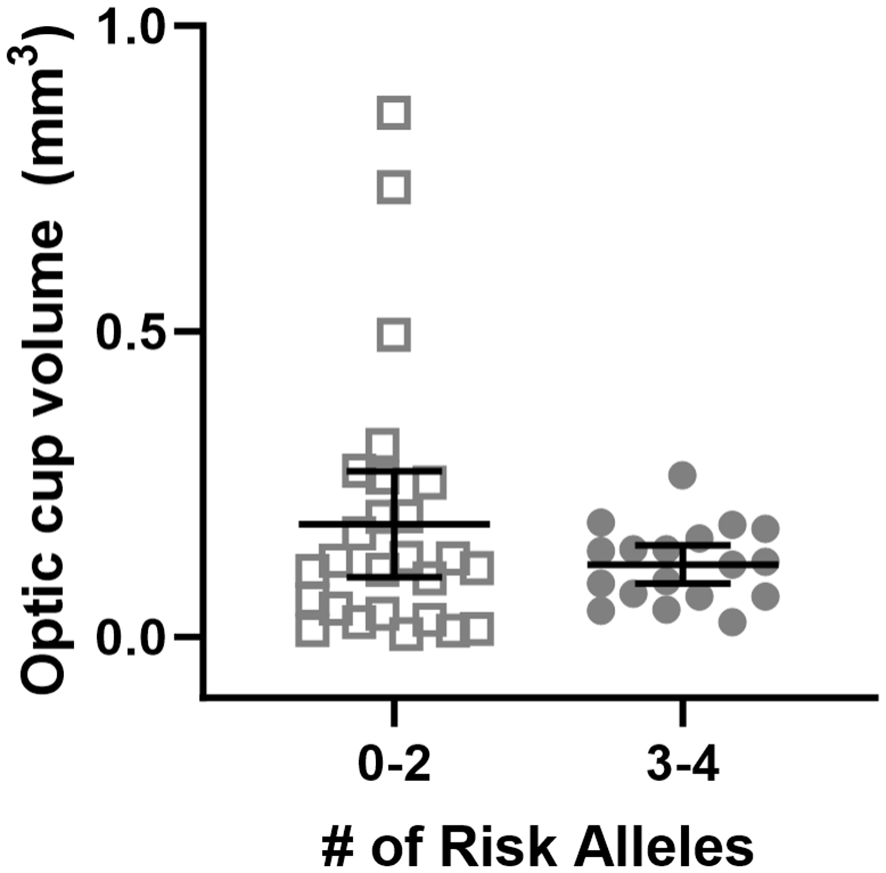
Baseline optic cup volume grouped by genetic risk allele category. Each data point represents a single eye. Optic cup volume was significantly different between genetic groupings where subjects were divided into those with 0-2 or 3-4 risk alleles for MTRR 66 and SHMT1 1420 (p<0.001).

Subjects with 0-2 risk alleles had larger baseline optic cup volume than subjects with 3-4 risk alleles (**Figure 2**, p<0.001). There is an interaction between genetic group and cup volume, indicating that having 3-4 risk alleles along with having smaller optic cup size is associated with a larger ΔTRT than the 0-2 risk allele group (p<0.001).

The subjects with 3–4 risk alleles in the previous 30-d HDTBR + 0.5% CO_2_ study, which was conducted in the same facility with the same standardizations (7, 8, 19), had a greater ΔTRT after 30 days of HDTBR (**Figure 1**, upper left panel) than the AGBRESA subjects with 3–4 risk alleles (**Figure 1**, upper right panel). The subjects with 1–2 risk alleles had similar ΔTRT in both studies.

In the group with 0-2 risk alleles, 2 of 11 (18%) had chorioretinal folds, whereas 4 of 5 (80%) had chorioretinal folds in the group with 3-4 risk alleles (p<0.001, **Table 1**). The estimate of the probability that the 3-4 allele group has a higher probability of total folds than the 0-2 allele group is 94%.

**Table 1:** Number of subjects with and without peripapillary, retinal, or choroidal folds after 60 d of HDTBR.

	0-2 Alleles	3-4 alleles
**Total n without folds**	**11**	**5**
**Total n with any folds**	**2**	**4**
Peripapillary folds	1	3
Retinal folds	1	2
Choroidal folds	0	2

The estimated posterior probability that subjects with 3-4 alleles have higher risk than those with 0-2 is 94%.

There were no differences in serum or red blood cell folate, vitamin B_12_, homocysteine, or vitamin B_6_ between subjects with 0-2 and 3-4 risk alleles (data not shown). Folate and vitamin B_12_ status were higher after 60 d of HDTBR similarly across genetic groups (p<0.001).

## 4 Discussion

These findings confirm that HDTBR subjects with more risk alleles have a greater ΔTRT (4, 5). The statistical model to predict ΔTRT improved when both genetics and optic cup volume were included. Although the increases in TRT are less for the AGBRESA study than the previous 30-d HDTBR study (7), the changes were significant in both studies; i.e., more G alleles for MTRR A66G and C alleles for SHMT1 C1420T correlated with greater ΔTRT during both studies.

We previously developed a multi-hit hypothesis describing how altered one-carbon pathway function could lead to SANS (20, 21). This study reinforces that there are likely multiple factors promoting optic disc edema during HDTBR in at risk individuals. We propose that genetic profile and optic cup volume are two such factors that together contribute to some of the variability in SANS presentation. Baseline optic cup volume has previously been shown to be a predictive factor for ΔTRT during spaceflight (3) but had not yet been assessed in bed rest or when combined with genetics. Here, we found that using both variables together improved the predictive model for the development of SANS findings, supporting the “multi-hit” aspect of the hypothesis. The relationship between SNP profile combined with anatomical variability of the optic cup needs further exploration, but the data reported here provide an explanation for further investigating how SNP profile may interact with the anatomical differences present at the optic cup that results in differences in ΔTRT despite all individuals having been exposed to the same headward fluid shift.

In this study, SNP category did not yield differences in serum vitamin biochemistry. Circulating vitamin status is not necessarily indicative of cerebral status, given they are required to not only be transported through the blood-brain barrier, but these vitamins are concentrated in the brain (22).

One of the main limitations of this study is the small sample size; however, the results support findings from a previous bed rest study (7). Also, the cause for the greater ΔTRT in the subjects with 3-4 risk alleles in the CO_2_ bed rest study compared to those with 3-4 risk alleles in the AGBRESA study is not known. While the participants in the CO_2_ study were exposed to 30 d of 0.5% CO_2_, they showed no change in arterialized and end-tidal PCO_2_ levels, cerebrovascular response to CO_2_, or hypercapnic ventilatory response (9, 10). Thus, without these other physiologic responses to increased ambient CO_2_ levels, it is difficult to assert that the differences in ΔTRT between the two studies are due to the CO_2_ exposure. Perhaps these differences are due to differences in subject selection (e.g., subjects all have different optic cup anatomy at baseline). Regardless, the same SNPs were associated with degree of optic disc edema in both bed rest studies.

We report here findings that further support a predisposition for some individuals to develop SANS. Work is ongoing to determine whether providing required cofactors (i.e., B vitamins) in the one-carbon metabolic network will improve ocular outcomes in at risk astronauts. Having a better understanding of the relationship between nutritional biochemistry, genetics, and optic cup volume may help to predict those at risk for SANS, and will improve our ability to provide targeted countermeasures for crew embarking on future exploration-class missions.

## Data availability statement

The OCT datasets for this study can be found in the NASA Life Science Data Archive 239 (https://nlsp.nasa.gov/explore/lsdahome).

## Ethics statement

The studies involving humans were approved by NASA Institutional Review Board and the Medical Association of the North Rhine (Aerztekammer Nordrhein). The studies were conducted in accordance with the local legislation and institutional requirements. The participants provided their written informed consent to participate in this study.

## Author contributions

SZ: Conceptualization, Formal Analysis, Funding acquisition, Methodology, Supervision, Visualization, Writing – original draft. BM: Conceptualization, Methodology, Writing – review & editing. SL: Conceptualization, Methodology, Visualization, Writing – review & editing. CF: Formal Analysis, Writing – review & editing. CS: Formal Analysis, Writing – review & editing. AS: Formal Analysis, Writing – review & editing. MM: Conceptualization, Writing – review & editing. MY: Data curation, Formal Analysis, Writing – review & editing. EB: Conceptualization, Writing – review & editing. SS: Conceptualization, Funding acquisition, Investigation, Resources, Writing – review & editing.
